# Adaptive Multi-scale Prognostics and Health Management for Smart Manufacturing Systems

**Published:** 2016

**Authors:** Benjamin Y. Choo, Stephen C. Adams, Brian A. Weiss, Jeremy A. Marvel, Peter A. Beling

**Affiliations:** 1 University of Virginia, Charlottesville, Virginia, 22904, U.S.A; 2 University of Virginia, Charlottesville, Virginia, 22904, U.S.A; 3 National Institute of Standards and Technology, Gaithersburg, Maryland, 20899, U.S.A; 4 National Institute of Standards and Technology, Gaithersburg, Maryland, 20899, U.S.A; 5 University of Virginia, Charlottesville, Virginia, 22904, U.S.A

## Abstract

The Adaptive Multi-scale Prognostics and Health Management (AM-PHM) is a methodology designed to enable PHM in smart manufacturing systems. In application, PHM information is not yet fully utilized in higher-level decision-making in manufacturing systems. AM-PHM leverages and integrates lower-level PHM information such as from a machine or component with hierarchical relationships across the component, machine, work cell, and assembly line levels in a manufacturing system. The AM-PHM methodology enables the creation of actionable prognostic and diagnostic intelligence up and down the manufacturing process hierarchy. Decisions are then made with the knowledge of the current and projected health state of the system at decision points along the nodes of the hierarchical structure. To overcome the issue of exponential explosion of complexity associated with describing a large manufacturing system, the AM-PHM methodology takes a hierarchical Markov Decision Process (MDP) approach into describing the system and solving for an optimized policy. A description of the AM-PHM methodology is followed by a simulated industry-inspired example to demonstrate the effectiveness of AM-PHM.

## 1. Introduction

In manufacturing, prognostics and health management (PHM) is growing as an alternative to reactive or fixed-interval policies for machine maintenance and replacement. Manufacturing PHM diagnostic and prognostic model leverages sensor data to estimate the health states of machines and their components, with these estimates often being expressed in terms of remaining useful life (RUL) (Lee et al., 2014). Health estimates are then the basis for deciding when to perform machine maintenance or replacement, so as to optimize costs and improve performance through the reduction of unplanned breakdowns. Such decision systems are adequate for use in legacy manufacturing environments in which operational profiles – such as machine cutting speeds, work cell production rate, and production line balance – are fixed or defined over narrow bands.

With the manufacturing world seeing an increase in automation and a greater inclusion of machines and robots within various processes (Marvel, 2014), global manufacturing initiatives are emphasizing the development and integration of smart manufacturing technologies. The smart manufacturing paradigm, which is seen as key to maintaining economic stability within an increasingly competitive global market (Holdren, 2011), includes sensing, communication, and computing systems that can support more dynamic control of operational profiles than is seen in traditional environments (Davis, Edgar, Porter, Bernaden, & Sarli, 2012). One may envision that next-generation PHM systems for smart manufacturing environments will use health estimates to inform control, maintenance, and replacement decisions based upon operational profiles.

Compared to traditional environments, smart manufacturing systems can be rapidly reconfigured to produce a new product, implement a new process, or take advantage of technological advancements in equipment. PHM decision systems to support smart manufacturing should share this agility by recomputing control policies on time scales that match the rate of change of the factory. The clear implication is that next-generation manufacturing PHM systems cannot be driven by historical observations of the production process alone, as the process will likely change before a model can be developed and leveraged.

In the manufacturing PHM literature, there is a notable absence of methodologies to support agile and flexible PHM systems in smart manufacturing environments (Peng, Dong, & Zuo, 2010). Additionally, the literature on manufacturing control does not address the use of health information in operational decisions beyond machine inspection, maintenance, and replacement (Choo, Beling, LaViers, Marvel, & Weiss, 2015). To address these gaps, we propose a methodology termed *Adaptive Multi-scale PHM* (AM-PHM). AM-PHM is characterized by its incorporation of multi-level, hierarchical relationships and PHM information gathered from a manufacturing system. AM-PHM utilizes diagnostic and prognostic information regarding the current health of the system and constituent components, and propagates it up the hierarchical structure. By doing so, the AM-PHM methodology creates actionable prognostic and diagnostic intelligence along the manufacturing process hierarchy. The AM-PHM methodology allows for more intelligent decision-making to increase efficiency, performance, safety, reliability, and maintainability.

AM-PHM, at a given level along the system hierarchy, receives operational directives and other constraints from the higher-level node. These operational directives and constraints describe the production goals under consideration by the decision makers (e.g., supervisors or planners) at the higher level. Based on the current health of the subsystems the decision maker (e.g., operators or control units) simulates the outcome of operating under several different modes of operation or operational profiles. The action that best fits the operational directive and constraints is selected. The expected results of the decision and the health state of the current node are reported to the higher-level node.

This paper takes a first step in demonstrating that the tradeoffs associated with PHM based decision making can be appropriately represented in a Markov Decision Process (MDP) framework. The contributions include a formulation of the AM-PHM methodology and a demonstration of the methodology on a simple example.

The remainder of the paper is organized as follows. Section 2 examines the current state of PHM capabilities and standards in manufacturing. Section 3 presents the concept of AM-PHM methodology. Section 4 includes the proposed AM-PHM features for describing the health state of systems in a hierarchical MDP framework and discusses example implementation of the AM-PHM methodology as applied to an industry inspired example work cell to show the effectiveness of the AM-PHM methodology. Section 5 concludes the paper by highlighting the significance of AM-PHM in manufacturing.

## 2. Current State of PHM in Smart Manufacturing

PHM is categorized into product PHM and process PHM depending on the nature of the system being monitored (Vogl, Weiss, & Donmez, 2014). Product PHM provides health monitoring, diagnostics, and/or prognostics for a finished system, such as an automobile, aircraft, or power generation station. Process PHM, on the other hand, provides health monitoring, diagnostics, and/or prognostics to a system that integrates one or more pieces of equipment to complete a task, such as in assembly processes, welding processes, and machining processes. The proposed methodology in this study develops a decision system for process PHM specifically for hierarchically structured manufacturing environments. However, the presented ideas and methodology could be extended to product PHM and other environments in future research.

Diagnostics, estimating the current health state of a system, and prognostics, estimating the future health state of a system, are essential for any PHM system. There is a significant amount of literature covering these subjects, including general reviews (Jardine, Lin, & Banjevic, 2006; Lee et al., 2014; Peng et al., 2010), method specific reviews (Si, Wang, Hu, & Zhou, 2011), and industry specific reviews (Hameed, Hong, Cho, Ahn, & Song, 2009; Lu, Li, Wu, & Yang, 2009; Sikorska, Hodkiewicz, & Ma, 2011; Zhang & Lee, 2011). However, these diagnostic and prognostic methods focus on accurately predicting current health states or remaining useful life (RUL), and do not address decision making in a sophisticated manner. If maintenance and replacement decisions are addressed in these studies, they are limited to a single action once the system has reached a critical threshold, and/or the scope is limited to a single machine or component.

Diagnostic and prognostic modeling occupies one region of the literature, while a separate region addresses PHM decision systems for manufacturing environments. The goal of a PHM decision system is to find maintenance and replacement policies using current and future health states, where a policy dictates actions given the health state or knowledge about the future health state of a system. A majority of the PHM decision systems assume that the health state information is given or can be found by inspecting the asset. Lam and Yeh (Lam & Yeh, 1994) compare five maintenance policies for a deteriorating system when the state of the system can be identified through inspection: failure replacement, age replacement, sequential inspection, periodic inspection, and continuous inspection. Grall *et al*. (Grall, Bérenguer, & Dieulle, 2002) consider both the replacement threshold and the inspection rate as decision variables. Both of these studies consider the system a single unit without any sub-components.

Numerous studies investigate multi-component systems under different assumptions about observing or estimating the health state of components. Multi-component decision systems often cluster or group maintenance activities under the assumption that the components are economically dependent (Bouvard, Artus, Berenguer, & Cocquempot, 2011). During group maintenance, several components are repaired at once, reducing overall cost. Shafiee and Finkelstien (Shafiee & Finkelstein, 2015) propose an age-based group maintenance policy on a multi-component system with two decisions: either the component has degraded to the point of replacement and preventative maintenance is performed on all components in the system, or the entire system undergoes preventative maintenance at a given time even if none of the system’s constituent components have worn to the point of replacement. Van Horenbeek and Pintelon (Van Horenbeek & Pintelon, 2013) propose a method for maintaining multi-component systems where the current state is found through inspection, and the RUL is then estimated based on the current state. The proposed methodology in this study assumes that the state information is available to the decision system. However, AM-PHM could be easily adjusted to include inspection to gain state information.

There are several studies that implement decision making using diagnostic and prognostic estimations of the health state (Montgomery, Lindquist, Garnero, Chevalier, & Jardine, 2006; Wu, Tian, & Chen, 2013; Yam, Tse, Li, & Tu, 2001). These studies consider the entire system as a whole with a single estimate for the health of the system, and do not address the fact that the system is composed of several sub-components which influence the health state estimates. Jonge *et al*. (de Jonge, Klingenberg, Teunter, & Tinga, 2016) combine the idea of clustering maintenance for multi-component systems with diagnostic monitoring of the component health states.

All of the previously referenced studies on PHM decision systems limit the scope of decision making to a single level. Nguyen *et al*. (Nguyen, Do, & Grall, 2015) investigate a multi-level decision-making maintenance policy, where the two levels are the system level and the component level. Huynh *et al*. (Huynh, Barros, & Berenguer, 2015) also develop a multi-level maintenance policy for complex systems and use a *k*-out-of-*n*:F deteriorating system where *k* components must fail in order for the entire system to fail. Inspection reveals the current state of the component, then RUL is estimated given the current state. While these two studies expand decision making to two levels, this is far from considering the entire hierarchy of a manufacturing system when making maintenance and replacement decisions. To the best of our knowledge, there is no literature addressing PHM decision systems for hierarchically structured manufacturing systems that consider decisions multiple levels above the component level.

Markov decision processes (MDPs) are a widely used model for decision making and have been applied to the maintenance and replacement decision making process. An MDP is composed of states, actions, and a reward signal, where a reward is received for taking an action given a state: and then the state evolves. In an MDP the next state of the system is determined by the current state and action. The history as to how the current state has been reached does not affect the system’s transition into the next state. Thus, an MDP is an effective way of representing a manufacturing system in which the next state of production or wear is dependent upon the current state of production or wear and the action taken. A detailed description of an MDP is presented in a later section. Amari *et al*. (Amari, McLaughlin, & Pham, 2006) use an MDP for finding an optimized policy for maintenance on a single diesel engine, and Robelin and Madanat (Robelin & Madanat, 2007) apply MDPs to bridge deck maintenance. Chan and Asgarpoor (Chan & Asgarpoor, 2006), Tomasevicz and Asgarpoor (Tomasevicz & Asgarpoor, 2009), and Chen and Trivedi (Chen & Trivedi, 2005) use semi-Markov decision processes for PHM decision systems. A semi-Markov decision process is an extension of an MDP where the duration spent in states is modeled. Maillart (Maillart, 2006) explores partially observable MDPs (POMDPs) as a non-specific PHM decision system, and Byon *et al*. (Byon, Ntaimo, & Ding, 2010) and Byon and Ding (Byon & Ding, 2010) both apply POMDPs to wind turbines. POMDPs are another extension of MDPs where the state is not observable, but signals correlated with the hidden state are observable.

Studies on MDPs and PHM decision systems are limited to single entities due to the curse of dimensionality (Powell, 2011). For MDPs, the curse of dimensionality refers to the increased difficulty in 1) estimating the value of actions given the state and 2) finding optimal policies as the state and action spaces increase in size. Therefore, the literature regarding MDPs and PHM decision systems often assumes that the possible number of health states is small, and that the possible number of actions is also small. In contrast, the methodology presented in this study proposes to model numerous components at multiple levels of a hierarchy with a large action space. A straightforward flat application of any of these methods to a system with several hundred components is difficult due to the explosion of the state and action spaces.

One assumption common to all the papers discussed so far in this section is that operations remain constant and are not a decision variable. For example, the cutting speed of a turning process is held constant and cannot be adjusted. AlDurgam and Duffuaa (AlDurgam & Duffuaa, 2013) propose a partially observable MDP in which operations are considered a decision variable. AlDurgam and Duffuaa represent changes in the operations through multiple transition matrices. The AM-PHM methodology will closely resemble the model in (AlDurgam & Duffuaa, 2013), but with multiple components and a completely observable state space.

In summary, the existing literature on PHM decision systems does not consider the entire hierarchy of the manufacturing environment. A vast majority of the literature focuses on single components or machines, and studies which do consider multi-component systems do not go past what this study would consider the machine level. The literature regarding MDPs as PHM decision systems is limited to small state and action spaces due to the curse of dimensionality, and the significant increase in the number of computations required to find an optimal policy. A vast majority of the literature assumes that operations remain constant and are not considered a decision variable. The AM-PHM methodology will address these limitations in the existing literature.

## 3. Adaptive Multi-scale PHM Concept

Existing literature on PHM decision systems is limited to maintenance, replacement, and inspection decisions, and lacks consideration of operational decisions. Also, the literature focuses on either components or groups of components, and does not consider work cells or assembly lines. Furthermore, most literature is based on unrealistic assumptions, such as a small state spaces, or consistent operations over time. AM-PHM contributes to the world of process PHM by addressing these limitations through a novel integration of health information into operational decision-making.

AM-PHM is a methodology that enables intelligent control for hierarchical manufacturing systems. It is designed to provide decision-makers with values for adhering to policies, the current health state of assets in the manufacturing system, and the predicted health states of assets at future points in time. In AM-PHM decisions are made in a sequential manner at each decision node. Production order information and operational directives are passed down the hierarchy, and component or machine health information is passed up the levels of the hierarchy.

In AM-PHM, a decision-maker is not limited to the machine operator. Rather, it refers to any person or machine such as the control unit of a manufacturing robot or the supervisor of an assembly line that is responsible for making decisions that can influence the outcome of the system. Each node of the hierarchical structure is a decision point where the decision-maker is situated in the AM-PHM methodology. Conceptually, an AM-PHM module resides at every decision point of the hierarchical structure of the manufacturing system.

Once all information is gathered at the decision nodes, AM-PHM creates operational profiles, which include operational policies and projected health information. Each operational profile is associated with a value for following a policy. Estimated by AM-PHM, a value is a cumulative reward for following a profile. Decision-makers, whether it be a person or a machine, can then choose the operational profile best suited for completing requirements and directives. Conceptually, decisions at the highest level can be thought of as a multi-objective reward function, where the goal is to maximize, through a sequence of decisions, a set of weighted rewards

(1) $$\text{max}\sum_{k=1}^{K}\omega_{k}R_{k}$$

where *K* is the number of objectives, *R_k_* is the reward associated with the *k^th^* objective, ω_*k*_ is the weight given to the *k^th^* objective, 0 ≤ ω_*k*_ ≤ 1∀*k*, and $\sum_{k=1}^{K}\omega_{k}=1$.

In AM-PHM, the decision making person at the highest level selects the weights, and then passes them down to the lower levels of the hierarchy where the objective function is used to find an optimized policy. Decision-makers select weights based on the current objectives of the manufacturing facility, such as meeting regular demand, meeting unexpectedly high demand, or planning for a lack of demand in the future. Multi-objective functions are passed down from the highest level to lower levels, where the lower levels are modeled as smaller separate models. The lower level system reports back the optimized likely outcomes based on the particular reward structure, objective function, and constraints handed down by the higher-level node.

**Figure 1:**
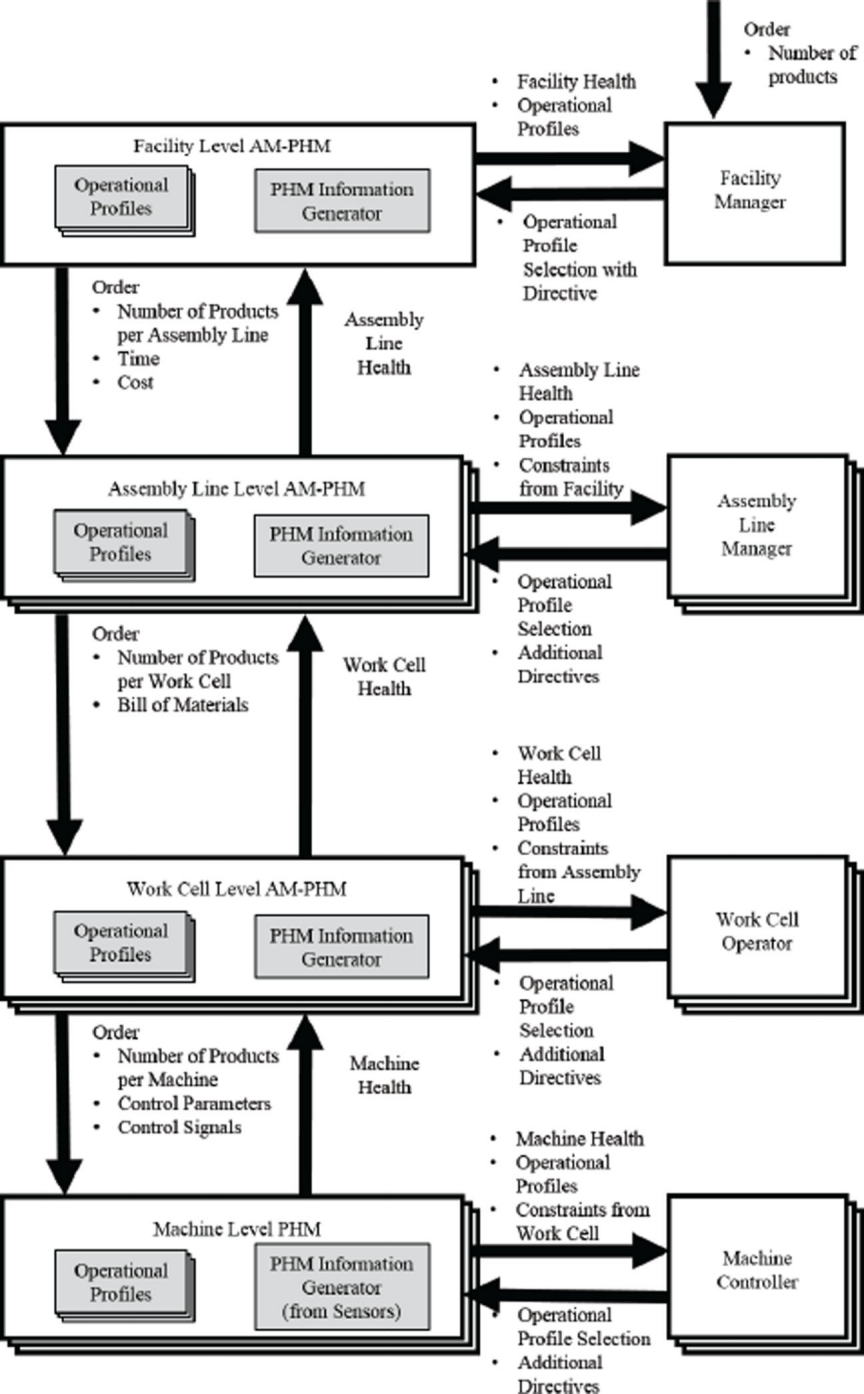
Conceptual representation of AM-PHM

To form an optimization problem which can be solved, the conceptual information flow such as in Figure 1 must be converted into a model. AM-PHM formulates a mathematical model by representing the system in terms of the decisions, states of the system, and rewards. Actions including maintenance decisions and operational policies are determined by optimizing for a multi-objective reward structure, which can change based on the production goals and directives sent from the higher levels of the hierarchy. Thus, AM-PHM addresses the task of model formulation, multi-scale decision making, and hierarchical health information formulation.

AM-PHM formulates the model by converting the conceptual information flow displayed in Figure 1 to mathematical models with objective functions that can be solved to find policies. Specifically, this task involves defining the inputs and outputs of the model; defining the time scale and time horizon at each level of the hierarchy; defining how health information flows up the levels of the hierarchy and how objective functions flow down the levels of the hierarchy; and outlining mechanisms for creating single reward functions from multi-objective functions from higher levels of the hierarchy.

AM-PHM addresses the decision making at each level of the hierarchy. In AM-PHM, decisions begin at the highest level because this is where the company defines their productivity and quality targets. Decisions flow downward into specific machining processes and equipment demands. As a machine’s health degrades, that information flows upwards and impacts decisions related to productivity. Given these decisions, multi-objective functions are developed and passed to the lower levels. A major portion of this task involves converting high-level decisions into multi-objective functions that can be optimized at lower levels.

A manufacturing system is often represented as a hierarchical structure. For a typical manufacturing facility there are assembly/fabrication lines which are further divided into work cells or work stations which are further divided into one or more machines (Hopp & Spearman, 2011). For this paper, the hierarchical structure of facility, assembly line, work cell, machine, and component will be used as a primary example, although more complex methods of describing a manufacturing facility exist.

In AM-PHM, health information is passed up the hierarchy. The health state at each node is an abstraction of the health state of its subsystems. The health state at a node may be represented as a vector of all the health states of the subsystems, the weighted average of the health of the subsystems, the worst health among the subsystems, or as a result of a function that accounts for the interconnected relations of the subsystems as necessary to convey health information.

One notable assumption made at this point for AM-PHM is that the health model for the lowest level of the hierarchy is known and the health states of the lowest subsystems are accurately known. The AM-PHM methodology also assumes that the hierarchical structure of the manufacturing system is known. The issue of not having an accurate model of the machine or component is discussed in Section 5.

## 4. AM-PHM Description

In this section a mathematical framework for describing the AM-PHM methodology is presented. One of the trade-offs that the decision-maker must keep in mind is to weigh the immediate reward(s) versus long term reward(s). An MDP-based approach in modeling the manufacturing system is one way to find an optimized decision that considers such tradeoffs. In an MDP model the transition to the next state depends on the current state and not on the history of state changes leading up to the current state. For a manufacturing system the next wear state of a component depends on the current wear state and not so much as to how the component reached its current wear state. Thus, an MDP approach is appropriate for describing the manufacturing system and the AM-PHM methodology.

However, the MDP-based model has complexity issues that make the description of a large scale system challenging. Hence, a hierarchical MDP approach is introduced. The hierarchical MDP approach divides the system into smaller more manageable sub-sections such as the machine or component model. The hierarchical MDP approach of the AM-PHM methodology is applied to an industry inspired numerical example.

### 4.1. Markov Decision Process

The framework used for the AM-PHM methodology is to describe the manufacturing system as an MDP. The reason for using an MDP approach is that in the MDP framework the next state of the system depends on the current state and not on the history of how the current state was reached. For example, the critical information needed to predict the wear of a component at the next sampling point is the current wear on the component and how that component will be used.

There are four elements that make up the MDP model - *state, transition probability, action*, and *reward*. The state space 𝒮 is a finite set of states that the system can be in. Actions 𝒜 is a finite set of actions that are possible in the system. State transition probability ℘, and reward ℛ are defined in Equations (2), and (3), respectively. (Sutton & Barto, 1998)

(2) $${℘}_{ss\prime }^{a}=\text{Pr}(S_{t+1}=s\prime |S_{t}=s,A_{t}=a)$$

(3) $${ℛ}_{s}^{a}=\{R_{t}=r|S_{t}=s,A_{t}=a\}$$

where the state of the system at time *t* (*S_t_*) is state *s*, the action taken at time *t* (*A_t_*) is *a* and the system transitions into state *s*′. The transition probability matrix ℘ is the probability of the state changing from one state to another depending on the current state s and the chosen action *a*. For an action *a*, each row of ℘^*a*^ represents the current state and the columns correspond to the next state. Thus, element ${℘}_{s,s\prime }^{a}$ represents the probability of the system transitioning from state *s* to state *s*′ when action a is taken. Since, the ℘ is a collection of probability distributions, the sum of each row of the sub-matrix ℘^*a*^ must add up to 1.

The reward ℛ is the reward associated with the system being in current state *s* and taking action *a*. The reward reflects a cost straucture that is of interest to the stakeholder such as the objective in Equation (1). The objective is now to maximize the expected cumulative reward.

A policy π is a mapping of states to actions and the distribution of possible actions given the state is π(*a*|*s*). A major component of solving an MDP is finding the value of being in a state. For MDPs, the value of a state and following a policy is defined as υ_π_(*s*) = 𝔼[*G_t_*|*S_t_* = *s*]. In an MDP the state transition is stochastic meaning that the state and action only selects a probability distribution for state transition. Therefore, the value of being in a particular state consists of the reward for the current state and the expected reward from the next states. A state action pair when following a policy can also have a value, which is defined as *q*_π_(*s, a*) = 𝔼_π_[*G_t_*|*S_t_* = *s, A_t_* = *a*]. The value function and the action-value function can be decomposed into

(4) $$\upsilon_{\pi }(s)=\sum_{a\in 𝒜}\pi (a|s)({ℛ}_{s}^{a}+\gamma \sum_{s\prime \in 𝒮}{℘}_{ss\prime }^{a}\upsilon_{\pi }(s\prime )),$$

and

(5) $$q_{\pi }(s,a)={ℛ}_{s}^{a}+\sum_{s\prime \in 𝒮}{℘}_{ss\prime }^{a}\sum_{a\prime \in 𝒜}\pi (a\prime |s\prime )q_{\pi }(s\prime |a\prime ).$$

where γ is the discount factor which is a number between 0 and 1 used to discount the reward received in the future. For this paper γ is set to one, meaning there is no discount. The MDP is solved by finding the policy that maximizes the value function or the action-value function. For example, in small MDPs, with less than several dozen states, the optimal policy π can be found through backward dynamic programming. For MDPs with large state and action spaces, iterative methods must be used to find a policy.

### 4.2. AM-PHM as MDP

The MDP model approach is used to formalize the AM-PHM methodology. In the AM-PHM world, since the expected future reward for a system depends on its current health state and not on the previous steps it took to reach the current health state, the MDP approach is the appropriate framework for representing the manufacturing system. Another notable characteristic of system health is that the health states do not transition in reverse, simplifying the transition matrix. A worn-out component will not turn back to a newer state unless a maintenance action is taken. The components have a probability of transitioning to a more worn-out state depending on the operation decision made by the decision-maker. Such a characteristic helps simplify the state transition property in the MDP.

The decision-maker now has a tool to quantitatively compare the case of immediate fast production causing greater wear on components versus the case of slower production leading to lesser wear on the components. A simple one level MDP description is given as an example. The hierarchical approach will be addressed in subsequent subsections.

(6) $$𝒮=\{s_{1},s_{2},\ldots ,s_{n}\}$$

(7) $$𝒜=\{a_{1},a_{2},\ldots ,a_{m}\}$$

The state space 𝒮 is the finite set of states used to describe the manufacturing system. In AM-PHM the states are based on the health of the system as in Equation (6) where *s_k_* represents a particular health state such as *Good, Ok*, or *Worn*.

The action space 𝒜 is the finite set of actions that can be taken by the decision-maker as in Equation (7) where *a_k_* corresponds to a possible action such as *Fast, Slow*, or *Run Maintenance*. Note that the action provided is not limited to a maintenance decision but includes operational decisions as well.

The transition probability matrix ℘ is the probability of the state changing from one to another depending on the current state *s* and the chosen action *a* as shown in Equation (2). ℘ can be built from historic operational and maintenance data, or from machine models.

The reward ℛ in Equation (3) indicates the amount of reward associated with the current state *s* and action *a*. ℛ is built from historic cost and production information or the user may define ℛ as deemed appropriate. The reward structure is also called the cost structure in some cases. The objective of the MDP is to create a policy that will maximize/minimize a partiuclar cumulative function of the reward. Thus, the reward structure may change according to the user’s directives.

### 4.3. AM-PHM as Hierarchical MDP

In theory, the transition matrix ℘ needs to cover all possible transitions between states. The size of ℘ is *mn*^2^ at the lowest level description of the system. The reward function ℛ needs to cover all the states and actions. The resulting ℛ is an *n* × *m* matrix for the lowest level description of the system. When the MDP framework is expanded to describe multiple components and machines, the number of possible states increases exponentially which also increases the size of the transition matrix exponentially. The size of the transition matrix for a manufacturing system consisting of *x* identical work cells, each containing *y* identical machines, each containing *z* components is shown in Equation (8). If each work cell structure is heterogeneous, the number of states will be the product of all the states of the lowest level nodes.

(8) $$N(S)=n^{\text{xyz}}$$

The size of the transition matrix and the size of the reward function in the expanded model is shown in Equations (9) and (10), respectively.

(9) $$N(P)={\text{mn}}^{2\text{xyz}}$$

(10) $$N(R)={\text{mn}}^{\text{xyz}}$$

The size of the state space and the transition matrix grows exponentially as components are added into the system. The size soon becomes too large to handle. Also, in reality the number of observable transitions between states is limited. It is difficult to observe or explore all possible state transitions to fill the entire transition matrix. For example for a manufacturing system with five states per component, four components per machine, four machines per work cell, four work cells per assembly line, two assembly lines per facility and three possible actions at the lowest level, the transition matrix will have 3 × 5^(2×4×4×4×2)^ elements which is greater than 2:59 × 10^179^. Thus, a more manageable approach is needed.

The flat MDP approach where no hierarchical structure is assumed, runs into the curse of dimensionality (Powell, 2011) as machines are added into the model. Even if the operations are limited to two modes, the action space quickly grows with the number of components or machines. Similarly, the state space, which represents the health of all components or machines, also quickly grows as components and machines are added to the hierarchy. Methods are needed to break this curse of dimensionality and, at least in an approximate or heuristic sense, compute policies for large state and action spaces.

One method to overcome the exploding dimensionality is the hierarchical MDP approach. By utilizing the hierarchical information of the manufacturing system the state space is reduced to a more manageable level. Most manufacturing systems are structured in a hierarchical manner. If the hierarchy of the system is known, then the system may be divided into smaller sub-MDPs.

Three approaches to hierarchical MDP were independently developed around the turn of the century: the options formalism (“Between MDPs and semi-MDPs: A framework for temporal abstraction in reinforcement learning”, n.d.), the hierarchy of abstract machines (Parr & Russell, 1998), and the MAXQ framework (Dietterich, 2000). Each of these treats the root problem at the top of the hierarchy as a semi-Markov decision process (Sutton & Barto, 1998) because sub-tasks can take a variable length of time. The options method breaks the problem into options that include a policy, a termination condition, and an initiation set of states. The hierarchy of abstract machines method breaks the problem into independent automata called abstract machines that include a set of machine states, a function for converting the states of the whole MDP into machine states, and a stochastic next-state function. The MAXQ framework decomposes the value function and considers each sub-task an MDP. In all three approaches, the hierarchy must be provided by the designer of the system.

In this research, the MAXQ framework will be adopted and customized to work with AM-PHM because a manufacturing facility can be easily broken down into sub-MDPs given its natural hierarchy. The MAXQ method can be transferred to the manufacturing environment by treating components at the lowest level of the hierarchy as independent and having their own state space. Further, the hierarchical decomposition allows for the policies learned on one sub-task to be transferred to other similar sub-tasks.

Dietterich (Dietterich, 2000) provides a list of five conditions under which one can reasonably implement state abstraction. This original set of five conditions is designed for hierarchical systems with sequential tasks. However, conditions such as requirements for a termination state for each sub-MDP can be relaxed for a manufacturing system with parallel assembly lines, work cells, or machines. The modified condition for state abstraction for the reduction of state space in a hierarchical MDP approach is reduced to three conditions. The first condition calls for the transition probabilities of the sub-MDPs to be independent. In a manufacturing system the state transition in one machine does not effect the state transition probability of another machine. The second condition calls for the reward structure of the sub-MDPs to be independent. The reward for taking action *a* in one machine is only affected by the current state of that particular machine and not by the current states of other machines in the manufacturing system. The third condition calls for the result distribution of a sub-MDP to be independent. For example with the existence of a smaller subset of funneling terminal states of a sub-MDP, other sub-MDPs are shielded from the sub-MDP having different starting states which allows for state abstraction. For a parallel manufacturing node the starting state of one sub-MDP does not have an effect on other sub-MDPs as each MDP is considered independent from the others.

The state space can be abstracted for a manufacturing system satisfying these conditions such as a parallel manufacturing system with a fleet of identical work cells or a sequential manufacturing systems separated by a buffer of work-in-progress parts. State abstraction reduces the state space to a more manageable level. The MAXQ approach is further developed into an online model-free version of the reinforcement learning algorithm called MAXQ-Q, where the value-action function is now dependent on the *i^th^* sub-task: *q*_π_(*i, s, a*) = υ_π_(*s, a*) + *c*_π_(*i, s, a*), where *c*_π_(*i, s, a*) is the expected discounted cumulative reward for completing subtask *i*.

### 4.4. AM-PHM Example

An example assembly line involving multiple machines is described in this section. The assembly line consists of two work cells -Work Cell 1 and Work Cell 2. Both work cells are comprised of two machines and produce identical products. Machines 1 and 2 are in Work Cell 1 and Machines 3 and 4 are in Work Cell 2. The machines can operate under two different modes of production - *Fast* and *Slow*. *Fast* production results in greater wear to the machine while achieving greater production than in *Slow* production. Assume that the health of Machine 1 is at 66% of its RUL while the health of Machines 2, 3, and 4 are at 100%. The assembly line only has enough resources to operate one machine for 60 days. The assembly line decision-maker must distribute the limited resources to the four machines and decide on the mode of production for each machine with the goal of achieving greatest profit. Assumptions and simplifications were made in this example scenario to prevent the example from becoming unnecessarily complicated. One of the assumptions is that the wear of the system is independent of individual operators. Nonetheless, the structure and the ratio of the cost and time involved are derived from real world manufacturing data.

The goal is to make a sequence of decisions or create a policy for making sequential decisions that will maximize the expected gain under the set of constraints. The decision space in the example manufacturing system is not limited to the maintenance decisions but is expanded into operating parameter decisions as well. Since the health state of the machines is either known or estimated, and the different modes of production lead to different frequencies in maintenance, the decision-maker needs a method for weighing the trade-off between short term gain and long term effects on system health in order to make an optimized decision. The AM-PHM methodology with the manufacturing system described as an MDP is an effective way to find this optimal policy. The numerical analysis in this section was performed in MATLAB using the Markov decision processes toolbox.

**Figure 2:**
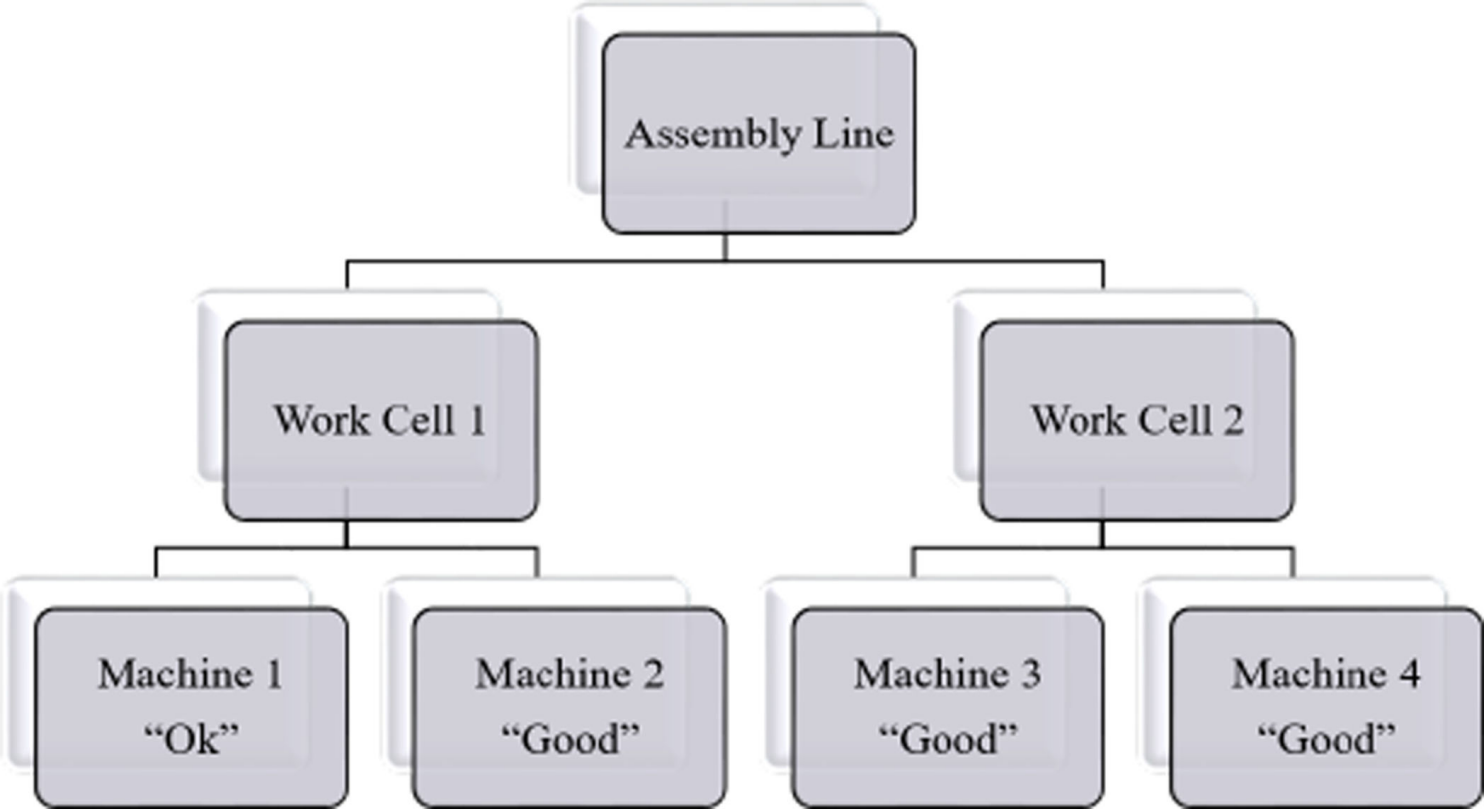
Example Assembly Line Hierarchy

In AM-PHM, existing knowledge on the hierarchical structure of the system is taken into account. The hierarchical approach allows for decomposition of a large scale model into a set of more manageable smaller size subsets and achieves agility by only having to partially reconstruct the model when sub-systems are replaced. The example hierarchical structure of a manufacturing environment used in this paper consists of a single assembly line with multiple work cells, each of which has multiple machines, each in turn comprised of multiple components. However for simplicity, the component level is not considered in this example as shown in Figure 2. The assembly line consists of two work cells. Work Cell 1 and Work Cell 2 each produce identical products and consist of two machines each.

The states reflect the health of the system. At the component level the wear progression trend of the component is known through wear curves, user experience, and material property based models. The discretization into health states is user defined. The states may be divided based on equal wear increments, equal RUL increments, or any other user defined set of rules. In the example the health states are divided into three states based on equal RUL increments from experience. There are two additional maintenance states added to distinguish preventative maintenance and reactive maintenance. Note that any maintenance conducted before the health of the machine reaches the Bad state will be considered preventative maintenance, whereas maintenance conducted at the *Bad* state will be considered reactive maintenance incurring a higher maintenance cost and longer maintenance time. The complete set of states is represented in Equation (11).

(11) S={Good,Ok,Worn,Prev−Maint.,React−Maint.}

Health states at higher-levels of the hierarchy are represented based on the health of its subsystems and effects between subsystems. The states at higher levels may be represented as a discretized abstraction based on user defined criteria of the health states of the subsystem or as a vector of the health states of the subsystem. For the example in this paper for higher-level nodes a maximum health approach is taken where the states are represented as the best health of its subsystem.

The decisions available to the decision-maker are different depending on the level along the hierarchy. The available set of decisions is based on user input, user experience, and other inherent limitations such as safety restrictions or a machine’s limited capabilities. For the example in this paper, the available decisions at the assembly line and work cell level are the allocation of resources such as available man-hour or raw materials to each subsystem. At the machine level the available decisions are as listed in Equation (12). Note that the operational parameters and the maintenance decisions are among the available decisions. For higher levels in the example case, decisions are the distribution of resources to its subsystems.

(12) A={Slow,Fast,Maintenance}

Once the states and actions are defined, the state transition probability matrix (*P*) is constructed. The transition probability is the probability of transitioning to another state based on the current state and action. The transition probability is constructed from information gained through means such as historic data and physical models. The probabilities are constructed bottom-up as the probability from the lowest level will affect the transition at higher levels. The mean time between failures of the machine and mean time to repair were used to derive the transition probability of the example. The probability distribution for state transition for the *Slow* action is based on the mean time between failures for the machine which is estimated as 21 days. The *Fast* action is approximately twice the speed resulting in a 50% reduction in the probability to remain in the same state. The lower left elements of the transition matrices for actions *Slow* and *Fast* are 0 since the machine health cannot improve on its own. When the *Maintenance* action is chosen, the state transitions to either *Preventative Maintenance* or *Reactive Maintenance* depending on the current state. The mean time between maintenance is estimated to be 10 days for *Preventative Maintenance* and 13 days for *Reactive Maintenance*. There is a 10% chance that the maintenance is not performed perfectly resulting in a state of *Ok* instead of *Good* after maintenance. Equations (13) through (15) represent the transition probability matrices used in the example. The rows represent the current states and the columns represent the next states.

(13) $$P^{\text{Slow}}=[\begin{array}{ccccc} 0.86 & 0.12 & 0.01 & 0 & 0.01\\ 0 & 0.86 & 0.08 & 0 & 0.05\\ 0 & 0 & 0.86 & 0 & 0.14\\ 0 & 0 & 0 & 1 & 0\\ 0 & 0 & 0 & 0 & 1 \end{array}]$$

(14) $$P^{\text{Fast}}=[\begin{array}{ccccc} 0.43 & 0.51 & 0.03 & 0 & 0.03\\ 0 & 0.43 & 0.47 & 0 & 0.1\\ 0 & 0 & 0.43 & 0 & 0.57\\ 0 & 0 & 0 & 1 & 0\\ 0 & 0 & 0 & 0 & 1 \end{array}]$$

(15) $$P^{\text{Maint}.}=[\begin{array}{ccccc} 0 & 0 & 0 & 1 & 0\\ 0 & 0 & 0 & 1 & 0\\ 0 & 0 & 0 & 0 & 1\\ 0.09 & 0.01 & 0 & 0.9 & 0\\ 0.063 & 0.007 & 0 & 0 & 0.93 \end{array}]$$

**Figure 3:**
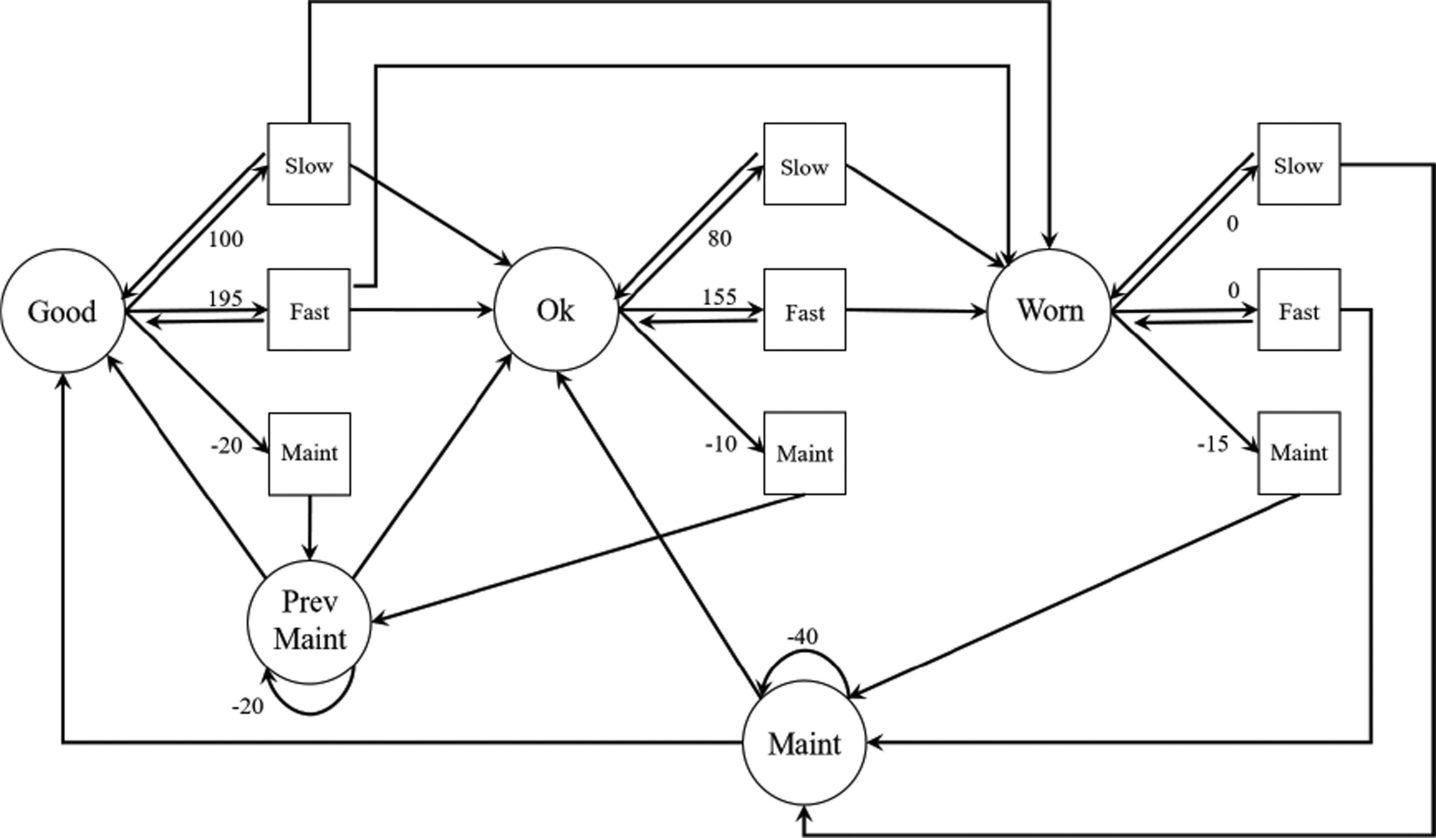
MDP of a machine with the state-action rewards marked.

The reward is assigned to each state-action pair and is defined by the user. Depending on the users focus, the reward may represent monetary cost and gain related to the system or may be the result of a scoring criteria created for the specific needs of the user. Which reward structure to choose is dependent on the goal set at the highest level of the hierarchy. The directive and constraints are handed down to the lower level nodes that specify which reward structure to choose. At the lowest level, the reward structure for this example is a combination of the cost of operation, the cost of maintenance, and the value added through production based on data obtained from an actual production facility. The system is optimizing for maximum monetary profit. At the higher-level nodes the reward is the sum of all the rewards from the sub-nodes. The health based states of the machine level and the associated reward are shown in Figure 3. For the reward matrix in Equation (16) the gains achieved by producing under *Slow* and *Fast* action are represented in the first two columns. The third column represents the reward associated with the *Maintenance* action. The bottom two rows of the first two columns are filled with a great loss to encourage a consistent selection of *Maintenance* action when in the maintenance states. The reward associated with the *Maintenance* actions are greatest when in the *Ok* state to highlight the fact that both premature replacement and reactive replacement cost more than replacing at an optimal point. The rewards are marked in the arrows in Figure (3). Note that rewards are only associated with state-action pairs. The actions *Fast* and *Slow* cannot be selected when in *Prev. Maint.* or *Maint.* state. When solving the MDP for the optimal policy, a negative number less than −40 was selected as the reward associated with these state-action pairs.

(16) $$R=[\begin{array}{ccc} 100 & 190 & -20\\ 80 & 150 & -10\\ 0 & 0 & -15\\ N/A & N/A & -20\\ N/A & N/A & -40 \end{array}]$$

**Figure 4:**
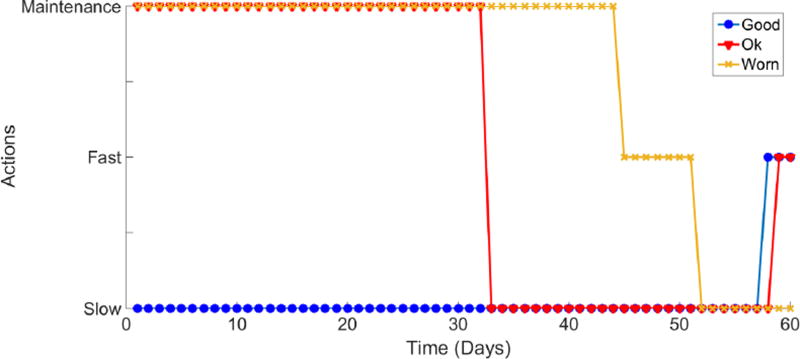
Policy for machine operation. The policy for each individual machine is identical.

Once the system is modeled as an MDP, the optimal policy search is performed. The goal is to create a sequence of decisions to follow depending on the current state that would maximize the cumulative reward over time. The operational directives and constraints flow from the higher-level node to the lower-level nodes. The PHM information is reported from the lower-level nodes up to the higher-level node. At each node, decisions are made based on the projected outcomes calculated within the constraints and available health information. Figure 4 shows the optimal policy for any machine for a 60 day finite horizon. The displayed policy is the same for each of the four machines because they are identical. Note that the time unit has been scaled into days. However, any other time unit such as seconds, minutes, or hours may be used as the decision interval. The choice to use days as the time interval in this case is to simplify the example.

The information from the machine level analysis in addition to the knowledge on the current state of health of the machines is used to make decisions at the work cell and assembly line level. Following the constraints of the example case, the assembly line must find the best way to distribute the resources to operate for 60 machine-days and the health state of Machine 1 is *Ok* and the health state of Machine 2, 3, and 4 is *Good*. The assembly line asks each work cell to report back the estimated reward when 1, 2, 3, …, 60 machine-days of resources are handed down.

Each work cell asks each machine to report the expected cumulative reward when operated under the optimal policy for 1, 2, 3, …, 60 days. Since we know beforehand that the four machines are identical, an MDP for the 60 day horizon needs to be solved only once and the results can be shared among the four machines. In a system with heterogeneous machines this benefit of reusing the MDP results will diminish. However, even under heterogeneous machine conditions the hierarchical approach still provides a modular solution that prevents the system state space from expanding exponentially.

The work cells report the best policy for the amount of allocated resources and the expected reward. For Work Cell 1 it would be a skewed result as Machine 1 is not in good health. For Work Cell 2 it will always be an even split of resources between the two machines as both machines have equal health.

**Figure 5:**
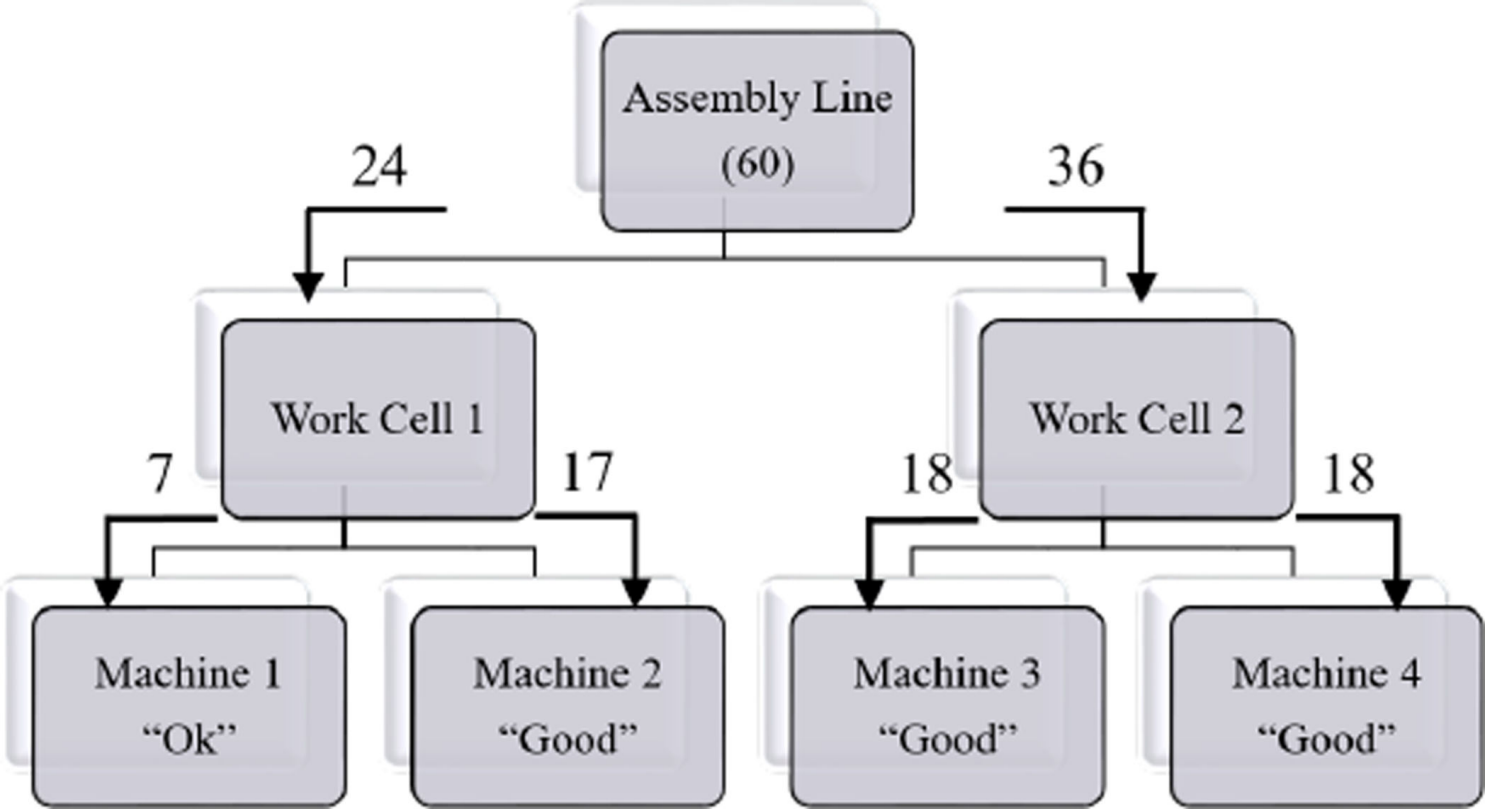
Resource distribution for the Assembly Line

The Assembly Line distributes the resources based on the best projected return from each work cell. In this example, the best distribution is to allocate 24 machine-days to Work Cell 1 and to give Work Cell 2 36 machine-days. Work Cell 1 will further distribute 7 machine-days to Machine 1 and 17 machine days to Machine 2. Work Cell 2 will split the resources evenly into 18 machine days and distribute to both Machine 3 and Machine 4 as shown in Figure 5.

AM-PHM is a methodology for constructing a dynamic control policy. Therefore, it uses the most up-to-date information when estimating the optimal policy. After one day has passed and resources have been initially distributed based on the previous policy, then the health states and the available resources are updated, and a new updated policy for optimal operation is derived using the current information. The process repeats until all 60 machine-day resources have been depleted.

## 5. Discussion

The AM-PHM approach suggests a way of making health information based operational decisions in a manageable way to increase operational efficiency. AM-PHM expands the use of health information from maintenance based decision making into operational decision making at all levels of the manufacturing system hierarchy. AM-PHM also takes a hierarchical approach to the analysis of the manufacturing system avoiding the issue of exponential state space growth associated with increase in the number of states, components, and machines. A simplified example MDP model of an assembly line based on data collected from an actual manufacturing facility demonstrates the usefulness of AM-PHM.

In representing the AM-PHM methodology as a hierarchical MDP, the hierarchical approach results in a collection of smaller, more manageable sub-MDPs compared to a flat MDP approach. Size reduction for the example case is from 5^(2×2)^ states for a flat MDP model to 5 states for the hierarchical MDP approach. The discrepancy between the flat model and the hierarchical model becomes more evident as more machines/components are added to the manufacturing system.

Several key assumptions were made for the hierarchical MDP representation of the AM-PHM methodology. First, we assumed that the health states of the machine/component were always accurately observable. Second, we assumed that not only the hierarchical structure was known beforehand but also the exact model for the individual machine/component was known. Third, we assumed that all the machines in the assembly line were identical, which made possible the repeated use of the same machine model. However, in reality some of these assumptions may be difficult to apply.

In the example, we assume that the transition probabilities of the MDP are given. In practice, these probabilities could be difficult or impossible to estimate from collected data for several reasons. First, readily available maintenance data could be difficult to map to the state transitions needed to construct the Markov chain transition probability estimates. Second, the amount of data needed to accurately estimate the transition probabilities grows significantly with the number of states. To address this issue, we propose using model-free reinforcement learning algorithms in future research on AM-PHM.

Reinforcement learning (Sutton & Barto, 1998) is the area of machine learning that deals with sequential decision making. In reinforcement learning, an agent or decision maker interacts with an environment, and based on the interaction receives a reward. The environment evolves with time influenced by the actions. An MDP is one model for the agent, environment, and reward often used in reinforcement learning. A policy is a function that maps states of the environment to actions, and learning this policy is the goal in sequential decision making. When the model for the environment is known, a policy is learned through planning. When the model is initially unknown, reinforcement learning is used to explore the environment and learn a policy. If the model cannot be adequately learned from interaction with the environment or the model is too large to solve efficiently, as is the case in most manufacturing facilities, model-free reinforcement learning algorithms should be implemented.

In reinforcement learning, a best policy may be derived without the full knowledge of the system model. However, there needs to be the possibility for the agent that is searching for the best policy to be able to interact with the target environment such as the machine. So if the agent has full access to a machine and is able to take different actions and learn from the reward and resulting state of the machine, then an optimal policy may be reached.

One challenge with reinforcement learning is that the agent must have access to the environment. However, in a manufacturing facility full access to a machine is not always granted. Thus, for future work we plan to build a machine simulator that will provide knowledge on the behavior of the machine to be used in the reinforcement learning process.

## 6. Conclusion

The AM-PHM methodology enables the creation of actionable prognostic and diagnostic intelligence up and down the manufacturing process hierarchy. Decisions are made with the knowledge of the current and projected health state of the system at decision points along the nodes of the hierarchical structure. To overcome the issue of exponential explosion of complexity associated with describing a large manufacturing system, the AM-PHM methodology takes a hierarchical Markov Decision Process (MDP) approach into describing the system and solving for the optimal policy. The AM-PHM methodology is applied to an industry inspired numerical example to demonstrate its effectiveness.

For future work, the AM-PHM methodology will be tested on data collected from an industry partner as well as being implemented on a production facility test bed. There are several challenges to the implementation that will be investigated in the future work. First, the MDP must be learned from collected data, however this could prove difficult because readily available maintenance data might not translate to MDP transition and reward functions. We propose constructing a simulation of the manufacturing environment to bridge the gap between the collected data and learning the MDP. Second, explore model-free reinforcement learning techniques that will learn policies through directly interacting with the simulation of the manufacturing environment. Third, in the example, it is assumed that system degradation is independent of individual operators. Future work will include an analysis of operator effect on the system. Fourth, it is assumed that the current health state and the remaining useful life of each component is known with certainty. Future work must integrate diagnostic and prognostic systems with the presented control system.

## Nomenclature

*K*: number of objectives
*R_k_*: reward associated with the *k^th^* objective
ω: weight given to the *k^th^* objective
𝒮: set of all possible states in the Markov Decision Process
𝒜: set of all possible actions in the Markov Decision Process
℘: state transition probability represented as a transition probability matrix in the Markov Decision Process
ℛ: reward associated with each state - action pair represented in matrix for in the Markov Decision Process
*S_t_*: state at time *t*
*s, s*′: states
*A_t_*: action at time *t*
*a*: action
*r*: reward
**Pr**(*X* = *x*): probability of random variale *X* being *x*
𝔼(*X*): expected value of random variable *X*
π: policy that defines the action to be taken for each state
υ_π_(*s*): expected total reward under policy π
γ: discount factor
*q*_π_(*s, a*): action-value function
*n*: number of states
*m*: number of actions
*N*(𝒮): number of states in state space 𝒮
*x*: number of work cells
*y*: number of machines for each work cell
*z*: number of components for each machine
*N*(℘): number of elements in the transition probability matrix ℘
*N*(ℛ): number of elements in the reward matrix ℛ
